# Longitudinal visibility of MRI findings in living victims of strangulation

**DOI:** 10.1007/s00414-024-03207-1

**Published:** 2024-04-02

**Authors:** Melanie Bauer, Christina Hollenstein, Johanna Maria Lieb, Sabine Grassegger, Tanja Haas, Laura Egloff, Celine Berger, Eva Scheurer, Claudia Lenz

**Affiliations:** 1 Institute of Forensic Medicine, Department of Biomedical Engineering, University of Basel, Pestalozzistrasse 22, Basel, 4056 Switzerland; 2 Institute of Forensic Medicine, Health Department Basel-Stadt, Basel, Switzerland; 3 Division of Neuroradiology, Clinic of Radiology and Nuclear Medicine, Department of Theragnostics, University of Basel Hospital, Basel, Switzerland; 4 Österreichische Gesundheitskasse im Gesundheitszentrum für Physikalische Medizin Liezen, Liezen, Austria; 5 Division of Radiological Physics, Department of Radiology and Nuclear Medicine, University of Basel Hospital, Basel, Switzerland

**Keywords:** Clinical forensic medicine, Forensic imaging, Living victims of strangulation, Magnetic resonance imaging, Head and neck imaging

## Abstract

Initial experiences with magnetic resonance imaging (MRI) of living strangulation victims demonstrated additional findings of internal injuries compared to the standard clinical forensic examination. However, existing studies on the use of MRI for this purpose mostly focused on the first 48 h after the incident. The aims of this study were (a) to evaluate the longitudinal visibility of MRI findings after violence against the neck by performing two MRI examinations within 12 days and a minimum of four days between both MRI scans and (b) to assess which MRI sequences were most helpful for the detection of injuries. Twenty strangulation victims participated in this study and underwent one (*n* = 8) or two (*n* = 12) MRI scans. The first MRI examination was conducted during the first five days, the second five to 12 days after the incident. Two blinded radiologists assessed the MRI data and looked for lesions in the structures of the neck. In total, 140 findings were reported in the 32 MRI examinations. Most of the findings were detected in the thyroid and the muscles of the neck. T_2_-weighted SPACE with fat suppression, T_1_-weighted TSE and T_1_-weighted MPRAGE were rated as the most helpful MRI sequences. Subjects who showed findings in the initial scan also demonstrated comparable results in the second scan, which was performed on average 8.4 days after the incident. Our results show that even up to 12 days after the incident, the criminal proceeding of strangulation cases may greatly profit from the information provided by an MRI examination of the neck in addition to the standard clinical forensic examination.

## Introduction

Various internal injury findings can be identified in the context of a fatal strangulation as demonstrated by forensic autopsies and indicated by postmortem imaging procedures. These findings may include for example hemorrhages or edema in various soft tissue structures of the neck, lesions of the intimal layer of carotid arteries or fractures of the laryngeal skeleton or hyoid bone [1–7]. However, the vast majority of internal findings have in common that they are not visible in clinical forensic examinations of surviving victims of violence against the neck [3, 8–12]. Therefore, the assessment and reconstruction of violent incidents in living persons with suspected strangulation is severely limited. Currently, strangulation is considered as dangerous to life if there are indications of hypoxia like unconsciousness, muscle tone relaxation or petechial hemorrhages, due to the congestion of the head following relevant and prolonged neck compression [13, 14, 3, 9, 15–18]. However, externally visible findings can be absent even in cases of severe strangulation or they may be visible for a time period of up to one or two days only [16]. This leads to unsatisfactory situations for the forensic assessment of relevant violence against the neck and the reconstruction of the incident. It further points out the necessity to assess additional objective findings, ideally during a prolonged period after the incident.

As initial experiences with magnetic resonance imaging (MRI) of living strangulation victims demonstrated, internal injuries can be identified in addition to the externally visible findings [8, 12–14, 17–20]. The internal injuries in locations directly related to the critical structures of the neck are thereby especially important, e.g. hemorrhages, localized edema and other lesions of vessels, lymph nodes, salivary glands, larynx, trachea, muscles and other soft tissue structures [14, 17–19, 21].

In most of the studies on MRI in living strangulation victims published so far, the main question was whether MRI can be used to prove relevant violence against the neck [8, 10–12, 14, 17, 18]. In 2007, Yen et al. [14] conducted the first MRI study on 14 strangulation cases and concluded that adding an MRI examination to the standard clinical forensic examination can generate additional insight by detecting findings, which would not have been visible otherwise. Moreover, in the study of Ogris et al. [12], a lack of correlations between external findings and internal findings identified through MRI was found. Therefore, the authors emphasized that only a clinical forensic examination in combination with an MRI leads to high sensitivity and specificity for the diagnosis of strangulation. Heimer et al. [11] published the study with the largest sample size so far. During six and a half years, they registered 633 living cases of strangulation of which 117 subjects underwent an MRI scan of the neck and the brain. The conclusion was that an MRI of the neck was beneficial for forensic purposes in cases without external findings, whereas an MRI of the brain should only be performed in cases with neurological symptoms. Pivec et al. [8] aimed at developing radiologic diagnostic criteria for the evaluation of living strangulation victims. As the inter-rater agreements of the two radiologists evaluating MRI scans were low to moderate, the authors concluded that experience in forensic radiology is necessary for an optimal evaluation. Furthermore, Christe et al. [17, 18] proposed a scoring system including MRI to assess findings pointing towards danger to life.

Summarized over all published studies, the most frequently reported internal findings were hemorrhages in the lymph nodes and in the muscles [8, 10–12, 14, 17, 18], while bruises and abrasions were the most frequent external findings [10–12, 17]. Furthermore, in all studies, the MRI examination was conducted on average within two days after the incident [8, 10–12, 14, 17, 18]. However, as in daily forensic practice the time between strangulation event and MRI appointment may exceed these two days due to a delayed reporting by the victim or MRI organizational issues, the question arises how long the internal findings remain visible. Therefore, the aim of this study was to evaluate the longitudinal visibility of MRI findings after violence against the neck by performing two MRI examinations for each victim with a time interval of at least four days between the scans.

## Materials and methods

### Study recruitment

This study was approved by the local ethics committee and all participants provided written informed consent. Participants were required to have experienced a strangulation event (regardless of type) that occurred less than 12 days prior to the first MRI examination and age of at least 18 years. The selection of the 12-day timeframe was influenced by the study of Yen et al. [14], where they detected findings in the neck MRI in a victim approximately 300 h after the strangulation incident. Exclusion criteria were general MRI contraindications, requirement of further medical treatment, arrest, departure from the country, presence of a language barrier and pregnancy (determined by a pregnancy test prior to each MRI examination).

Participants were recruited between 2019 and 2022 during the standard clinical forensic examination. Out of 42 subjects who were willing to take part, 19 people were excluded as they did not fulfill the inclusion criteria, withdrew their consent prior to the first MRI scan or were not reachable. Hence, MRI appointments were arranged with 23 study participants. Two candidates failed to appear to the agreed appointment and one candidate suffered from a panic attack due to claustrophobia during the scan. Consequently, those three were excluded from the study after initial enrollment. Thus, 20 victims of strangulation took part in this study and underwent one (*n* = 8) or two (*n* = 12) MRI scans.

### Standard forensic examination

The study participants underwent a standardized clinical forensic examination by a forensic pathologist conducted according to the guidelines defined by the Swiss Society of Legal Medicine (SGRM) [13]. The time between the strangulation incident and the clinical forensic examination was 0 to 2 days (mean ± standard deviation: 0.4 ± 0.6 days). The examination consisted of a thorough inspection of the whole body and especially the neck to detect injuries. The subjects were examined for any signs of strangulation or other violence such as scratches and intradermal or subcutaneous hemorrhages (i.e. bruises). Any findings, other information on the incident, as well as further symptoms, e.g. loss of urine, loss of consciousness or hoarseness, were thoroughly documented. The skin of the neck was documented with daylight photography and in 85% of the study participants additionally by infrared photography as an optional part of the institute’s standard clinical forensic examination [20, 22, 23]. As defined by the guidelines of the SGRM [13], concrete danger to life was attributed if signs of blood congestion of the head such as petechial hemorrhages of the mucous membranes of the eyes, mouth or on the skin of the head were present or if a loss of consciousness occurred.

### MRI examinations

If possible, two MRI examinations of the neck were performed within the timeframe of 12 days after the strangulation incident. The first MRI scan took place during the first five days (mean ± standard deviation: 3.0 ± 1.0 days), the second five to 12 days (8.4 ± 1.7 days) after the incident. The first and the second scan were four to seven days apart (6.2 ± 0.9 days). A minimum interval of four days was maintained between the MRI scans to ensure sufficient time for potential changes regarding findings between the first and second MRI scan. If the first MRI scan could not be performed due to time reasons, subjects only participated in the second MRI timeframe (*n* = 7). In one subject, only the MRI scan in the first timeframe was performed due to in the meantime launched Covid-19 restrictions. All MRI examinations were conducted using a 3 Tesla MRI system (Siemens MAGNETOM Prisma, Siemens Healthineers Erlangen, Germany) and a standard 20-channel head and neck coil with head fixation using foam insertions. The MRI acquisition covered the neck from the clavicles to the upper bony palate.

The protocol for all neck MRI scans included the following seven sequences without administration of intravenous or oral contrast agents:

- A coronal T_2_-weighted sampling perfection with application optimized contrasts using different flip angle evolution (SPACE) sequence with fat suppression (short tau inversion recovery; repetition time (TR) = 3200 ms, echo time (TE) = 198 ms, in-plane resolution = 0.4 mm^2^, slice thickness = 1.14 mm);
- A coronal T_1_-weighted turbo spin echo (TSE) sequence (TR = 700 ms, TE = 9.4 ms, in-plane resolution = 0.4 mm^2^, slice thickness = 3.0 mm);
- A transversal T_2_-weighted TSE Dixon sequence (TR = 8620 ms, TE = 82 ms, in-plane resolution = 0.4 mm^2^, slice thickness = 3.0 mm);
- A transversal T_1_-weighted TSE Dixon sequence (TR = 666 ms, TE = 12 ms, in-plane resolution = 0.4 mm^2^, slice thickness 3.0 mm, including an additional gradient echo with opposed phase for calculation of in-phase, out-of-phase, water and fat images);
- A sagittal 3D T_1_-weighted magnetization prepared rapid gradient echo (MPRAGE) sequence (TR = 1800 ms, TE = 2.77 ms, inversion time (TI) = 1000 ms, resolution = 0.5 × 0.5 × 1.0 mm^3^);
- A transversal diffusion weighted readout segmentation of long variable echo trains (RESOLVE) sequence (TR = 6700 ms, TE1 = 55 ms, TE2 = 95 ms, in-plane resolution = 1.2 mm^2^, slice thickness = 4 mm, b-value 1 = 50 s/mm^2^, b-value 2 = 800 s/mm^2^, 4 diffusion directions);
- A sagittal 3D fluid attenuated inversion recovery (FLAIR) sequence (TR = 5000 ms, TE = 280 ms, TI = 1800 ms, resolution = 0.5 × 0.5 × 1.0 mm^3^).

The total scan time was approximately 45 min. To minimize additional distress for already traumatized victims and in light of previous studies highlighting the limited benefits of brain MRI in strangulation victims, brain scans were not performed in this work [11].

### Radiological reading

Each MRI dataset was evaluated and interpreted independently by two board-certified radiologists. Radiologist 1 is specialized in neuroradiology as well as head and neck radiology and had a total work experience in radiology of 11.5 years at the beginning of this study without previous experience in forensic radiology. Radiologist 2 has a specialization in radiology, had three years of experience in forensic radiology and a total work experience in radiology of 14 years at study start.

**Fig. 1:**
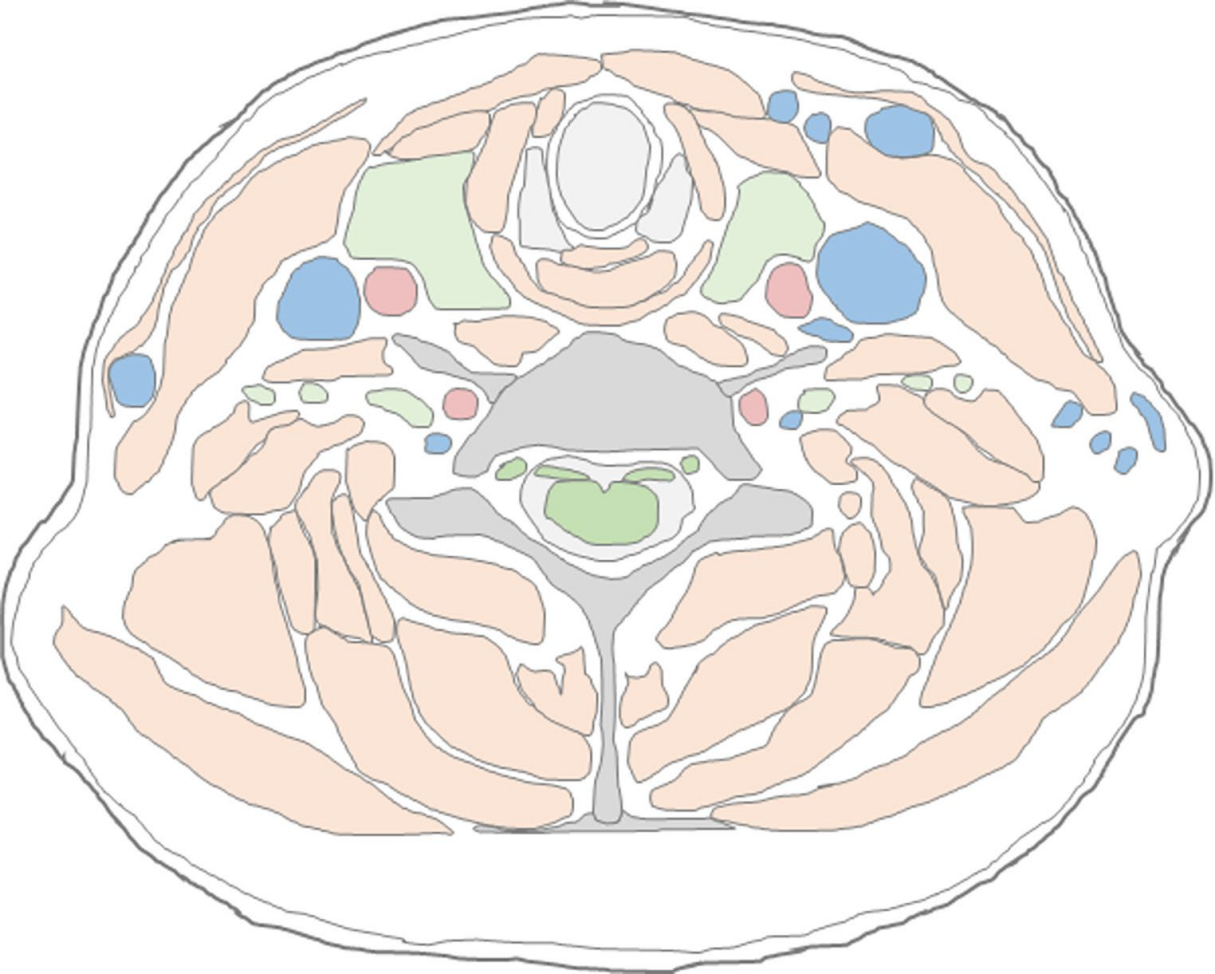
Exemplary transversal slice of the neck through the 6th cervical vertebra illustrating the rating scheme provided to the radiologists. (adapted from [24]). The anatomical structures are differentiated by colors

Both radiologists conducted the reading of the MRI data on a picture archiving and communication system (PACS) workstation and were blinded to any further information concerning the participant or the strangulation incident. Additionally, they did not know which scans originated from the same subject, as the first and second MRI scan were acquired with different study identifications. The radiologists were provided with a scheme of the neck divided into seven transversal slices of the neck, each slice through a different cervical vertebra [24]. For illustration purposes, one exemplary transversal slice of the scheme is shown in Fig. 1. The radiologists recorded each finding using the scheme and a detailed table (Microsoft Excel, Version 2002, Microsoft Corporation, Redmond, WA, USA) for each MRI scan separately. During the reading, the radiologists did not only look for hemorrhages and edema in the lymph nodes, muscles, salivary glands, thyroid, as well as in the cutaneous and subcutaneous tissue, but also for injuries of the blood vessels and the bone and cartilage structures of the neck. The radiological evaluation involved a stepwise approach: initially, asymmetries and size or shape variations in anatomical structures were identified, with diffusion data being crucial for larger entities like muscles and lymph nodes. This was followed by detecting hyperintensities in T_2_ SPACE data and hemorrhages in corresponding T_1_-weighted images. If there were any normal variations of anatomical structures, these were noted and described independently to the findings resulting from the strangulation incident. Incidental findings were communicated orally by the study coordinator to those study participants who had declared that they wanted to be informed at the time of inclusion to the study. Additionally, all study participants were provided with their MRI data upon request.

### Statistical analysis

Data curation was performed using Microsoft Excel (2016) and data processing was performed using Python (Spyder IDE 5.2.2 for Python 3.9.13). The interrater agreement of both radiologists was determined by calculating Cohen’s kappa. Resulting values equal or below 0 indicate poor agreement, between 0 and 0.2 slight agreement, between 0.21 and 0.4 fair agreement, between 0.41 and 0.6 moderate agreement, between 0.61 and 0.8 substantial agreement and between 0.81 and 1 almost perfect agreement [25]. Depending on the level of measurement, the significance of variable correlations was calculated with the Pearson correlation *pearsonr* (continuous data), the point biserial correlation *pointbiserialr* (continuous data - dichotomous or nominal data) or the Fisher exact test *fisher_exact* (nominal data – nominal data). The examined variables were: number of MRI findings, strangulation type, number of external findings, number of subjective symptoms and type of MRI finding (edema or hemorrhage). To test for significant changes of edema and hemorrhage sizes in the first and the second MRI scans’ data, the Wilcoxon signed-rank test *wilcoxon* was performed. A significance level of 0.05 was applied.

## Results

Of the 20 study participants (mean age and standard deviation: 33.1 ± 7.69 years, age range: 21–50 years), 15 were female and five male. 15 strangulations had been conducted manually. Equally often, one hand (right hand: *n* = 2; left hand: *n* = 1; unclear = 5) or two hands (from the front: *n* = 7; from behind: *n* = 1) were used. A forearm chokehold was performed in four cases and three victims were garroted (two with a jacket and one with a scarf). Two victims experienced both manual strangulation and a forearm chokehold. 11 study participants did know and eight participants did not know their offender. In one case, it was not clear whether the offender was known to the victim.

**Fig. 2:**
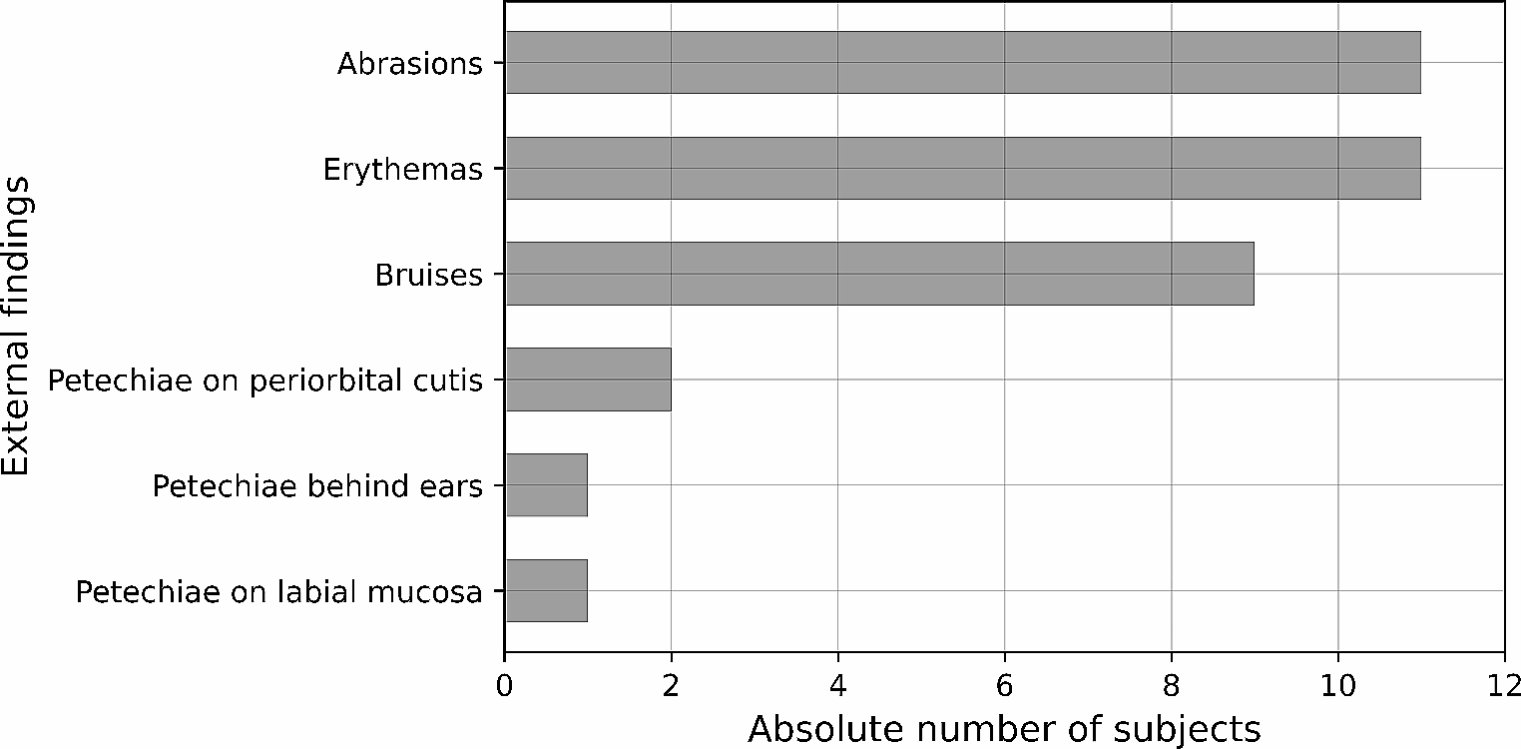
Categories of external findings detected in the standard clinical forensic examination of the 20 study participants. Multiple categories of findings per victim were possible

**Fig. 3:**
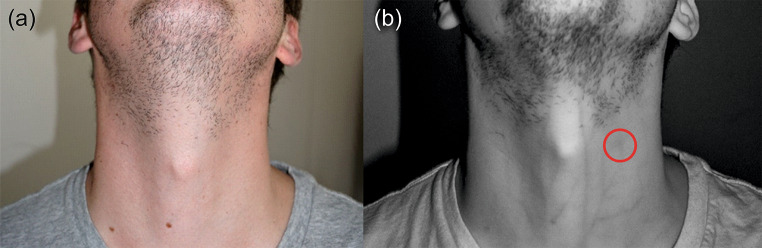
Exemplary photographic documentation of a strangulation victim. (**a**) Daylight photography with no visible findings. (**b**) Corresponding infrared photography showing a hematoma (red circle)

**Fig. 4:**
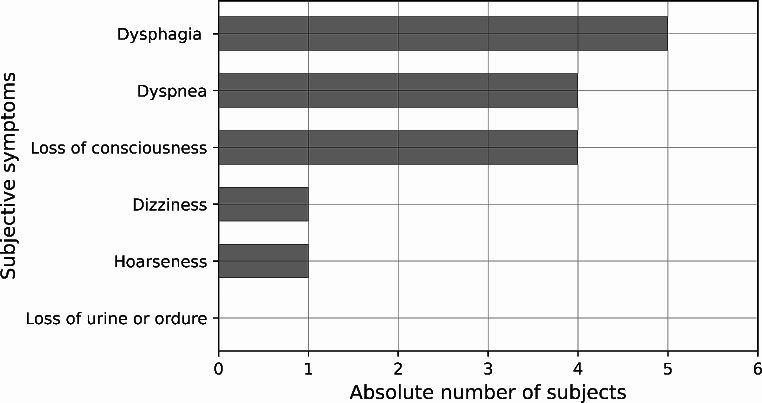
Categories of subjective symptoms detected in the seven out of 20 study participants who reported such symptoms. Multiple categories of subjective symptoms per victim were possible

An overview of the detected external findings is given in Fig. 2. The most frequently observed findings were abrasions and erythema (each *n* = 11), followed by bruises (*n* = 9). Five participants showed no external findings. The forensic pathologists detected petechial hemorrhages in three participants. As illustrated in Fig. 3, infrared photography revealed a hematoma in one subject who otherwise had no external signs. Seven participants reported subjective symptoms during the strangulation event including dysphagia, dyspnea and loss of consciousness (see Fig. 4). All subjects with reported loss of consciousness recovered spontaneously. In summary, a concrete danger to life was attributed to six study participants based on the standard forensic assessment.

**Fig. 5:**
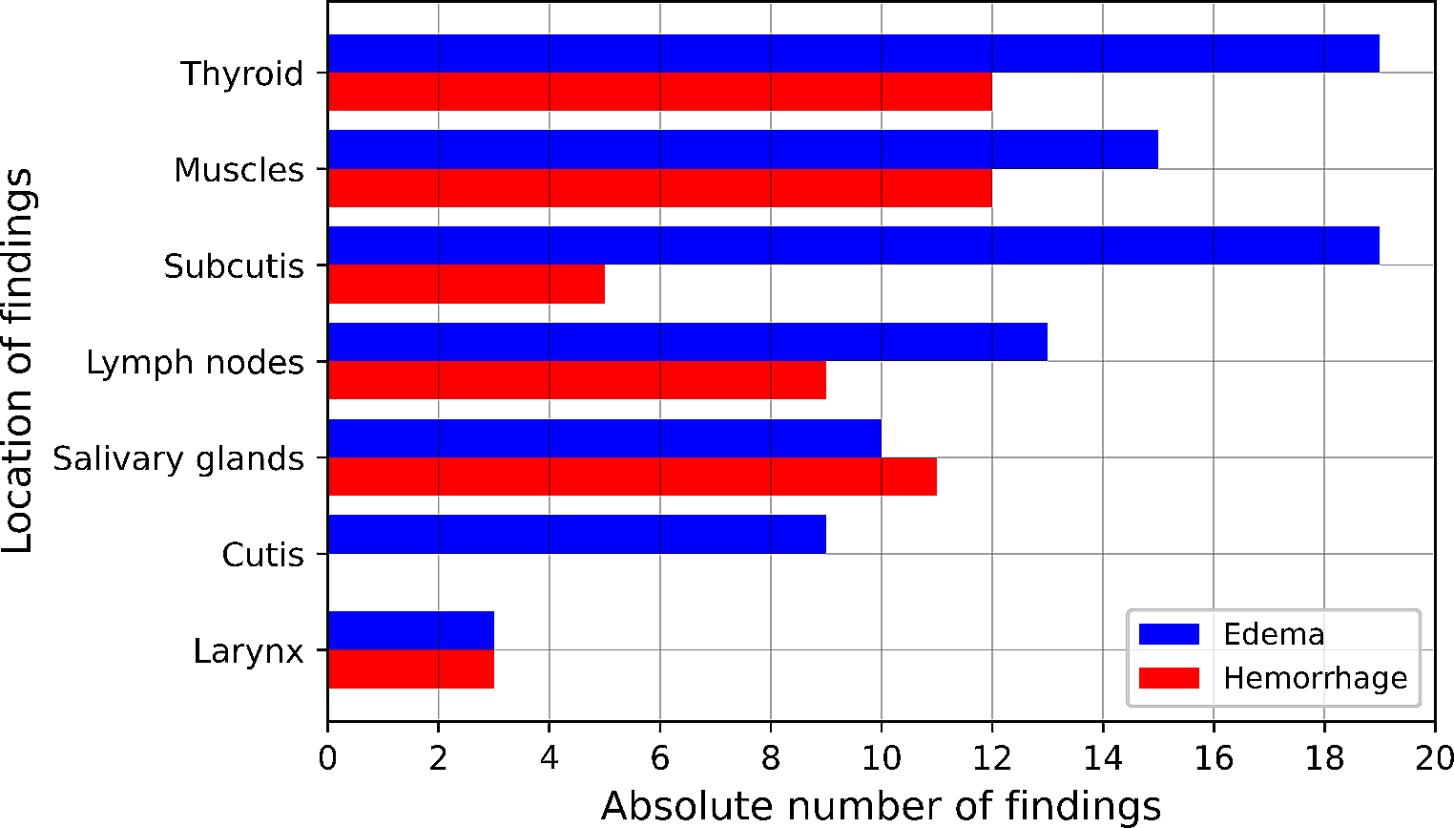
Locations of the 140 MRI findings from 32 scans of 20 subjects according to the type of finding (edema or hemorrhage) for both raters

**Fig. 6:**
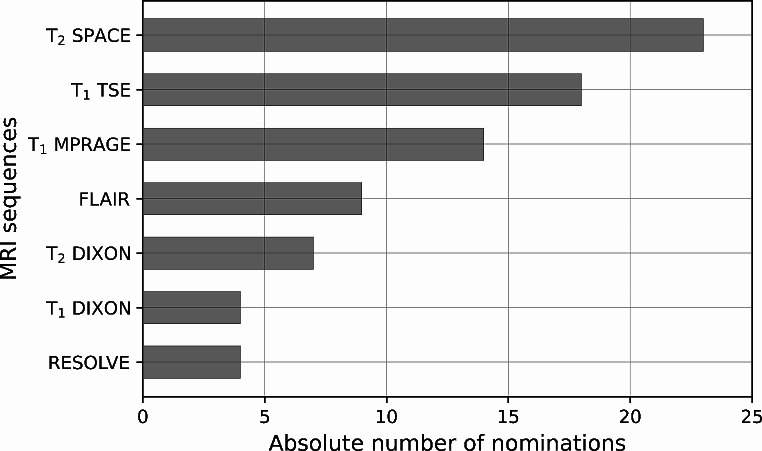
Usefulness of MRI sequences according to the subjective evaluation of the raters. Only MRI sequences with at least one nomination in the 32 MRI evaluations are shown. Multiple nominations of sequences per MRI scan were possible

A total of 140 MRI findings were reported by the two radiologists in all 32 MRI examinations. On average, the first MRI scan revealed 3.3 findings, while the second scan showed 4.6 findings. Across both scans, an average of 4.1 findings per scan and 6.5 findings per subject was detected. In two participants, no MRI findings were reported neither at the first nor at the follow-up scan. Of the 140 findings, only 7% (*n* = 10) were detected by both radiologists, while 25 (18%) were seen solely by radiologist 1 and 95 (68%) solely by radiologist 2, respectively. According to Cohen’s kappa, this result corresponds to a slight agreement (k = 0.01). The radiological findings are illustrated in Fig. 5 according to their location and type. The thyroid, muscles and subcutis showed the most findings, whereas no findings were reported for blood vessels and mucosal tissue. In total, edema occurred more frequently than hemorrhages (63% compared to 37%). Considering all MRI scans, 47 findings were detected in all examinations within timeframe 1 and 93 findings within timeframe 2. A significant correlation was found between the number of MRI findings and the strangulation type choke (*p* = 0.04). No significant correlations were detected between the number of MRI findings and the number of subjective symptoms (*p* = 0.15) and the number of external findings (*p* = 0.68), respectively. Besides, a significant correlation was found between strangulations using two hands and the presence of hemorrhages (*p* = 0.05). A ranking of the MRI sequences according to their usefulness in evaluating strangulation victims is shown in Fig. 6. The T_2_-weighted SPACE, the T_1_-weighted TSE and the T_1_-weighted MPRAGE sequences were rated as the most helpful by the radiologists.

**Fig. 7:**
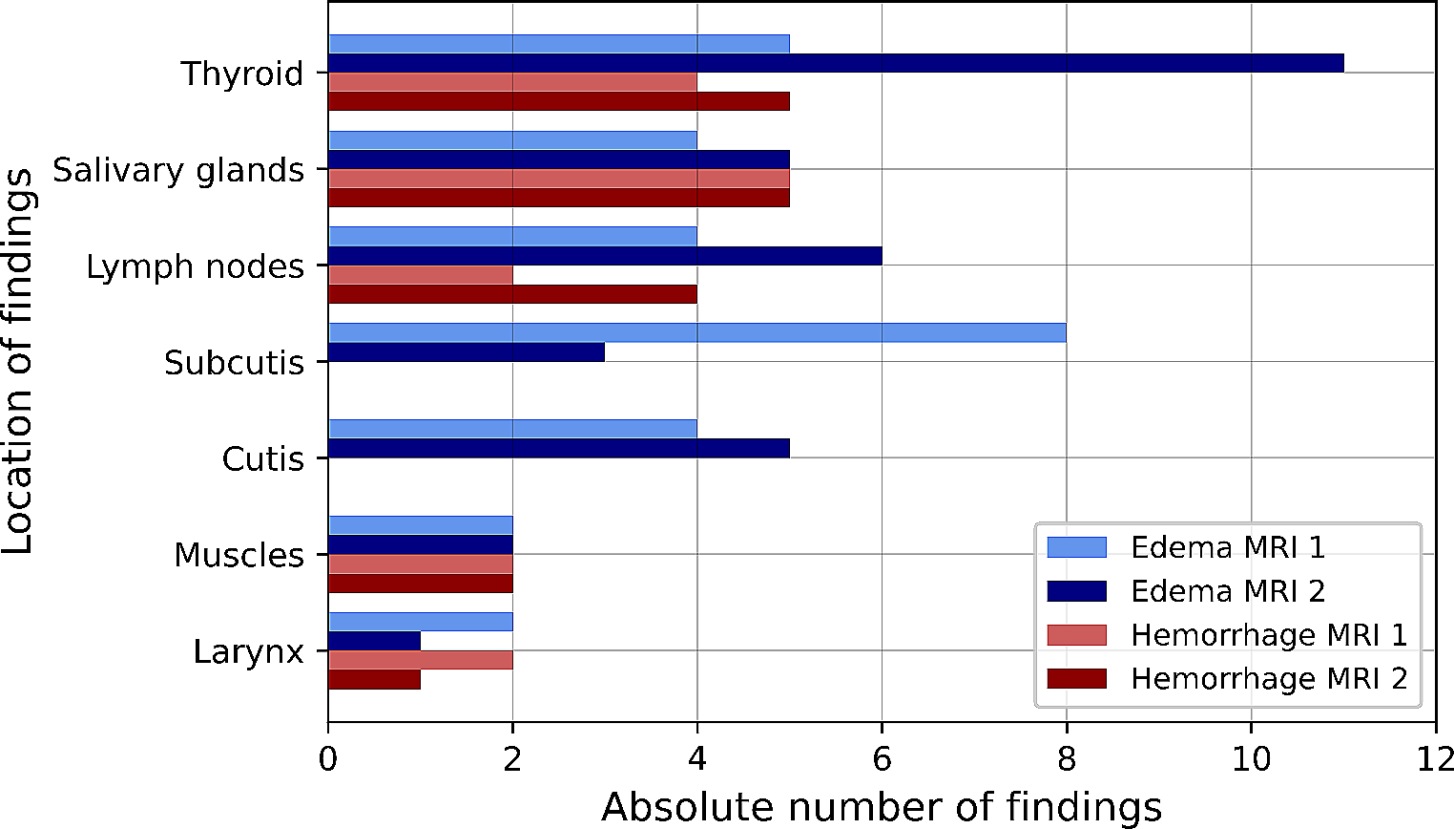
Locations of MRI findings in the 12 subjects who underwent two MRI examinations according to the type of finding (edema or hemorrhage) in the first (MRI 1) and second (MRI 2) timeframe. Multiple locations of findings were possible per subject

**Fig. 8:**
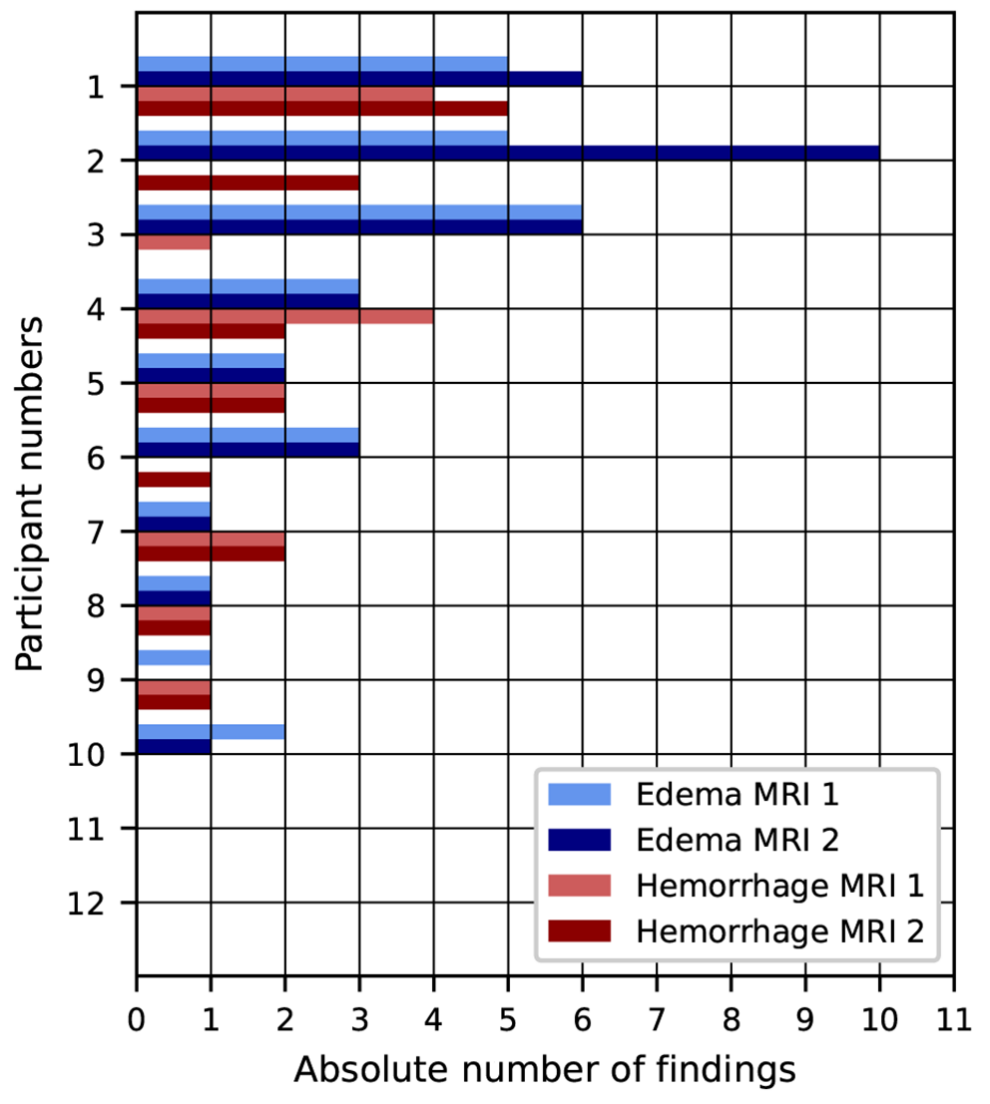
Findings of edema and hemorrhages for the 12 participants who underwent two MRI scans (for each participant and scan separately)

In the 12 participants who underwent two MRI examinations, 44 radiological findings were reported in the imaging data acquired within the first timeframe, and 50 findings within the second timeframe. There was no subject who showed radiological findings in the first MRI scan, but none in the second. The longitudinal results are presented in detail in Fig. 7 according to the location and type of the MRI findings. More edema findings could be seen in the subcutis and larynx in the first timeframe compared to the second. All other findings were still visible or even increased in number in the second timeframe. Figure 8 shows the changes between the first and the second MRI scan for every participant with two MRI scans separately. The number of edema decreased in two subjects and increased in two subjects at the second MRI scan, while six exhibited the same number of edema in both MRI scans. The number of hemorrhages decreased in two participants, increased in three participants and stayed the same in four participants. No statistically significant correlation was found between the number of newly detected findings between the first and second MRI scan and the time from the strangulation event to the first MRI scan (*p* = 0.20). In the MRI data of the first timeframe, the mean size of edema was 14.0 ± 10.6 mm, whereas the mean size of hemorrhages was 11.8 ± 12.5 mm. In the findings of the second timeframe, the mean size of edema slightly decreased to 13.4 ± 10.8 mm and the mean size of hemorrhages increased to 13.8 ± 12.0 mm, respectively. However, these differences were not statistically significant neither for edema (*p* = 0.20) nor for hemorrhages (*p* = 0.66).

**Fig. 9:**
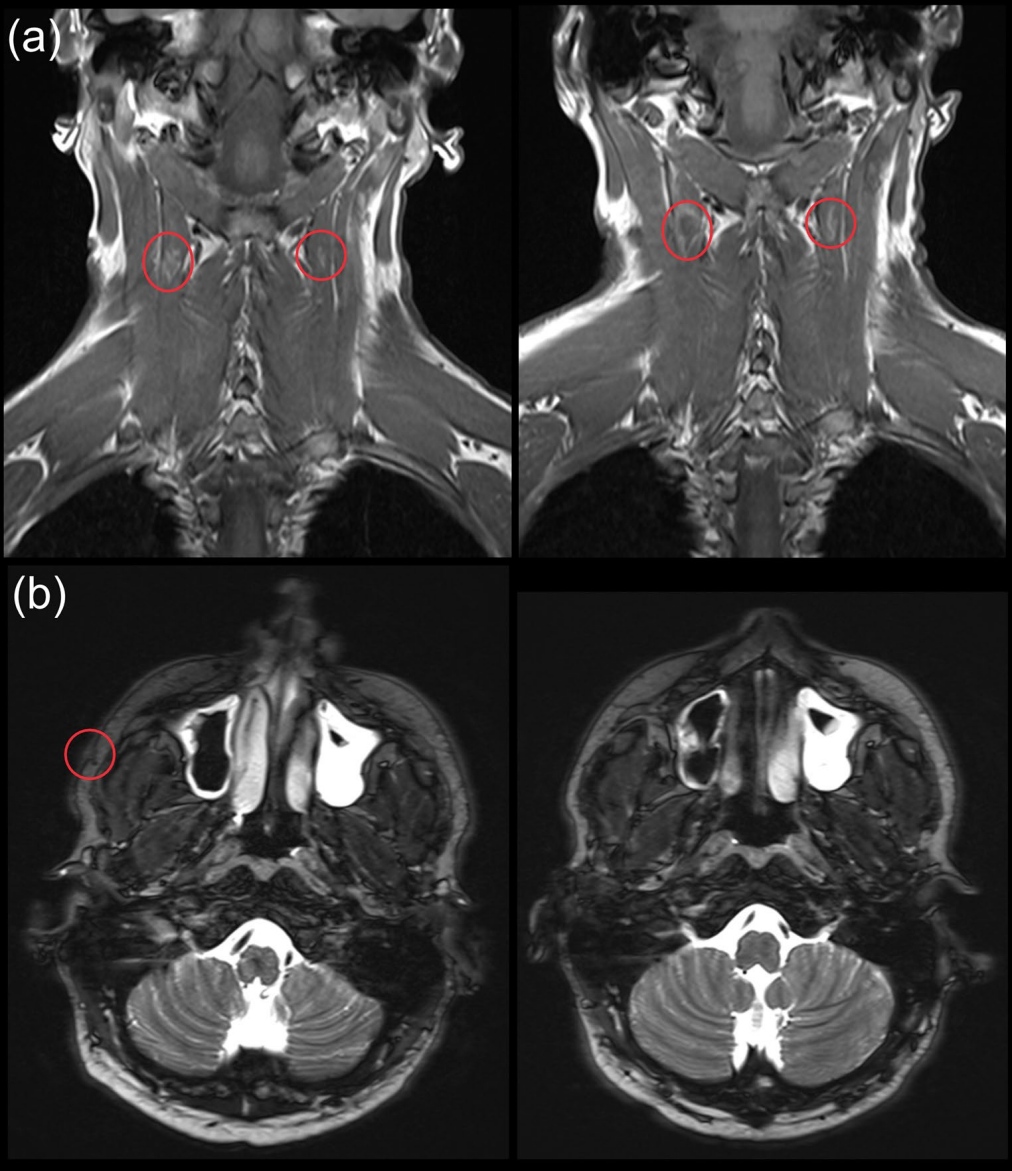
Exemplary MRI data. (**a**) Living strangulation victim showing MRI muscle findings in the coronal T_1_-weighted TSE sequence (red circles). Left: MRI scan acquired within timeframe 1 (4 days) after the incident, the muscle findings were rated as hemorrhages. Right: MRI scan acquired within timeframe 2 (11 days) after the incident, the muscle findings were still present and rated as edema. (**b**) Living strangulation victim showing an edema of the cutis in the transversal T_2_-weighted TSE DIXON opposed-phase MRI data (red circle). Left: MRI scan acquired within timeframe 1 (3 days) after the incident, the finding in the cutis was rated as edema. Right: MRI scan acquired within timeframe 2 (9 days) after the incident, the finding was no longer present (no red circle)

**Fig. 10:**
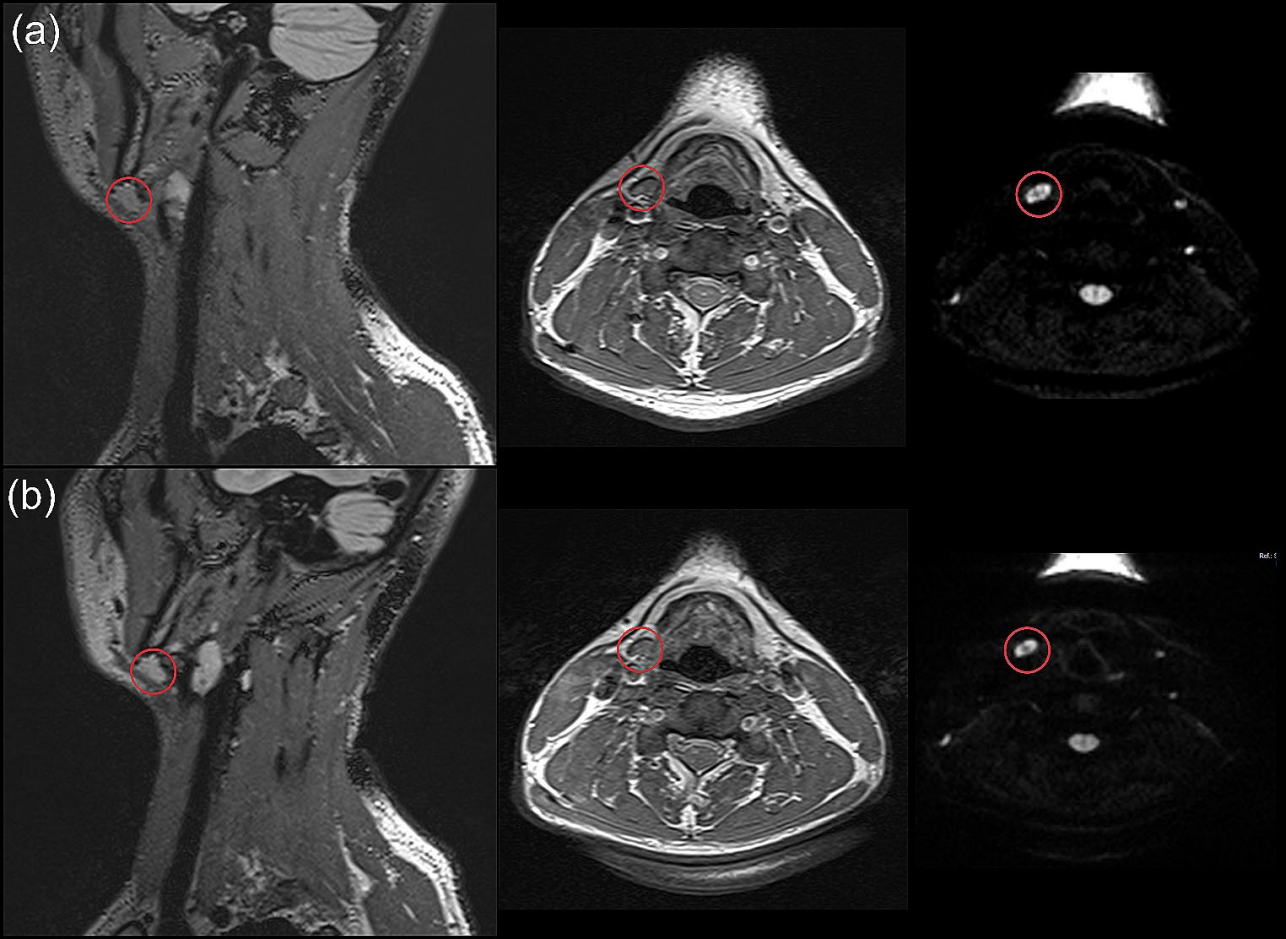
Exemplary MRI data of a living strangulation victim with no findings in digital photography, but a hematoma in the infrared photography (see also Fig. 3). In the MRI data, a hemorrhage of the cervical lymph node was detected in both MRI scans (red circle). (**a**) MRI scan acquired within timeframe 1 (3 days) after the incident, (**b**) MRI scan acquired within timeframe 2 (9 days) after the incident. Left: 3D FLAIR sequence (sagittal view); Middle: T_1_-weighted TSE DIXON in-phase data (transversal view); Right: trace-weighted RESOLVE diffusion sequence (transversal view)

Exemplary MRI data is presented in both Figs. 9 and 10. The findings in the muscle from a strangulation victim who reported to have been garroted are illustrated in Fig. 9 (a). These findings were still detectable in the second imaging timeframe, on day 11 after the incident. On the contrary, an edema of the cutis is shown in Fig. 9 (b). This edema had been resorbed between the two MRI scans. The radiological findings of the subject presented in Fig. 3 are further depicted in Fig. 10. This strangulation victim reported to have been strangled manually with both hands by an unknown aggressor. The participant showed a hematoma in the infrared photography, but no findings in the digital photography. Remarkably, a bleeding into a cervical lymph node was found in both MRI scans, which were performed on day 3 and 9, respectively, after the incident.

## Discussion

Several studies stated the importance of MRI for examining living victims of strangulation, as it can provide additional objective information on internal injuries of the neck [8–12, 14, 17, 18, 26, 27]. The goal of this study was to examine the longitudinal visibility of radiological findings in living victims of strangulation by performing two MRI scans of each participant.

In the 12 subjects who underwent both MRI examinations, it was shown that performing the first and the second MRI scan revealed similar results, even though the second MRI scan was performed on average 8.4 days and even up to 12 days after the incident of strangulation. Furthermore, no subject was reported with radiological findings in the first MRI scan, but no findings in the second one. Edema in the larynx and subcutis were absorbed quickly (before the second MRI scan), whereas edema in the thyroid, lymph nodes, cutis and salivary glands developed at a later time point (after the first MRI scan).

A reason for this could be that the edema in the thyroid, lymph nodes, cutis and salivary glands developed as a residuum after hemorrhage resorption, which takes time to develop after a strangulation event. In contrast, the low number of hemorrhages in the subcutis (*n* = 0), muscles (*n* = 2) and larynx (*n* = 2) limit the probability of developing such residua in these structures. Hemorrhages degraded in the larynx before the second MRI scan and developed even later in the lymph nodes and thyroid (after the first MRI scan). A study conducted by Hassler et al. [28] showed that MRI scans provide the means to measure the age of subcutaneous hematoma, as the degradation of blood follows a specific pattern. It is important to consider that on the one hand edema can develop late after the strangulation event, as a residuum of a resorbed hemorrhage and on the other hand hemorrhages can develop after an edema due to vessel damage by the increased pressure [29, 30]. The timing of edema and hemorrhage development varies individually and relies on factors such as the extent and duration of the strangulation, the physical condition of the victim and the location of the injury [31, 32].

No statistically significant differences regarding the mean size of findings in the first and second MRI scans were detected. Hence, on the basis of the results of this longitudinal study, we recommend to extend the possible timeframe for MRI examinations beyond the maximum of six days applied in previous studies [8, 10–12, 14, 17, 18, 27] to 12 days after the incident. This is especially important for forensic institutions with a lack of direct access to MRI systems, where scheduling of MRI appointments for strangulations victims may be difficult on short notice, and MRI scans need to be delayed due to fully booked clinical facilities. Additionally, also suspected victims of strangulation incidents who are reported with a delay of several days might profit from an MRI examination [16].

Our study participants showed external findings ranging from none to extensive bruises of the neck. The most common categories of external injuries in our study, i.e. abrasions, erythema and bruises, corresponded well with literature [33]. However, the number of external findings did not correlate with the number of MRI findings, with more findings found internally than externally. Thus, different conclusions could be drawn if the clinical forensic evaluation is performed without an “internal” assessment using MRI. Although infrared photography has not been used in all subjects, this technique can reveal hematomas despite the absence of findings on visual clinical examination or daylight photography. Additionally, it has demonstrated particular advantages when applied to heavily pigmented skin [23]. However, there is still a lack of studies focusing on its application to living victims [22].

MRI findings associated with the strangulation incident were reported in 18 of the 20 study participants. The most prominent findings were found in the thyroid and the muscles. In existing literature, findings in the muscle are described frequently [8, 11, 14, 17, 34]. However, less findings were detected in the thyroid in previous studies. This may be due to the high number of findings in the thyroid in the second MRI scans, which were performed up to 12 days after the incident, while existing literature performed MRI scans on average only within two days after the incident [8, 10–12, 14, 17, 18]. No fractures of the hyoid bone were found in this study [35]. This might be attributed on the one hand to the low resolution of MR images, the limited ability of MRI to visualize bones, the lack of bone displacement, absence of associated hemorrhage or edema around the fracture and on the other hand to the very rare occurrence of hyoid fractures in survivors of strangulation [3, 4, 35]. Notably, no internal findings were reported for a victim strangled with one hand who exhibited an erythema on the neck. Similarly, another victim who was garroted and experienced loss of consciousness, memory gaps, and intra- and subcutaneous bleedings, also had no discernible MRI findings.

In congruence with previous studies [33], the number of subjective symptoms reported by the victims did not correlate significantly with the number of MRI findings. This underlines that most symptoms highly depend on the subjective perception of the victim. However, the number of MRI findings correlated significantly with the strangulation type choke. Accordingly, Heimer et al. [11] stated that MRI may reveal internal findings in victims of chokes even if they do not show external injuries. Besides, using two hands for the strangulation correlated significantly with the presence of hemorrhages, which is a sign of stronger force to the neck.

Although both radiologists described various MRI findings, they had only a very small overlap corresponding to a slight agreement. This difference was not unexpected as interrater reliability is generally an issue in radiological studies, particularly when radiologists vary in their level and type of training [8, 11, 19, 21, 36]. In forensic radiological reading, forensic training of radiologists seems crucial, as clinical radiologists are not used to look for findings that are not clinically relevant, such as small hemorrhages or subcutaneous edema. The publications of Pivec et al. [8] and Heimer et al. [11] show a similarly low inter-observer agreement for MRI based strangulation findings between a forensically experienced radiologist and a clinical neuroradiologist without forensic experience. Therefore, as the radiological reading of the small and diffuse strangulation findings needs more diligence and a different approach than a standard clinical radiological assessment, it is recommended that the evaluation of MRI data of strangulation victims should ideally be performed by radiologists with training in forensic radiology or experience with forensic cases.

The extensive MRI protocol used in this study is based on sequences adapted for high quality imaging of the neck. The two radiologists appreciated the value of the T_2_-weighted SPACE with fat suppression, the T_1_-weighted TSE and the T_1_-weighted MPRAGE sequences. However, both radiologists also showed great differences in their subjective perception of the usefulness of the individual sequences, which is in line with the slight agreement of both raters for radiologic findings. In 2019, Heimer et al. [11] published the first study based on more than 100 cases, which was also the first study not recommending an MRI in every case of strangulation. As they also looked at the brain, the MRI protocol of the neck seemed to fall short and was not based on sequences optimized for the neck. It might be that this contributed to the fact that they found less than one finding per MRI scan, compared to up to 6.2 findings per MRI scan in our study, and concluded that an MRI should not be performed in every strangulation case. To maximize the output of an additional MRI examination in strangulation victims, it is highly recommended to set up an adapted MRI protocol of the neck, together with experienced MRI physicists, radiologists and radiographers and focused on edema and hemorrhages in soft tissue structures in order to benefit from technically optimized sequences.

This study demonstrates the benefit of an additional MRI examination compared to the standard clinical forensic examination and photographic documentation alone, as the MRI data provided additional information on internal injury of the neck.

Based on the results presented here, we agree with previous studies [8–10, 12, 14, 17, 18] that MRI should become a standard part of the forensic assessment of living strangulation cases. MRI seems especially beneficial when the clinical forensic examination results in no findings, as it is currently the only way of detecting injuries in deeper soft tissue and skeletal structures of the neck. Considering how difficult a strangulation case can be from a juridical point of view, the additional inclusion of MRI findings might help to enhance the overall assessment of strangulation cases, which is based on many different aspects, e.g. the forensic expert report, police reports and other results of the criminal investigation.

## Conclusion

In summary, we recommend the application of MRI in living victims of strangulation up to 12 days after a strangulation incident. Especially in victims without any external findings, further evaluation of internal findings based on MRI is beneficial. We further suggest the use of an extensive, state-of-the-art MRI protocol optimized for the neck and reading of MRI data by radiologists with experience in forensic radiology.

## Data Availability

The datasets generated during and/or analyzed during the current study are available from the corresponding author on reasonable request.

## References

[1] Jankovich L, Incze J (1933). Blutungen in den Lymphknoten Des Halses Beim Erhängungstod. Dtsch Z Gesamte Gerichtl Med.

[2] Saukko P, Knight B (2015) Knight’s Forensic Pathology, 0 edn. CRC Press.

[3] Yen K, Thali MJ, Aghayev E (2005). Strangulation signs: initial correlation of MRI, MSCT, and forensic neck findings. J Magn Reson Imaging: JMRI.

[4] Gascho D, Heimer J, Tappero C, Schaerli S (2019). Relevant findings on postmortem CT and postmortem MRI in hanging, ligature strangulation and manual strangulation and their additional value compared to autopsy - a systematic review. Forensic Sci Med Pathol.

[5] Deininger-Czermak E, Heimer J, Tappero C (2020). Postmortem Magnetic Resonance Imaging and Postmortem Computed Tomography in ligature and Manual Strangulation. Am J Forensic Med Pathol.

[6] Maxeiner H (1985). [Hemorrhages in internal laryngeal soft tissues]. Beitr Gerichtl Med.

[7] Fineron PW, Turnbull JA, Busuttil A (1995). Fracture of the hyoid bone in survivors of attempted manual strangulation. J Clin Forensic Med.

[8] Pivec SM, Scheurer E, Fischer F (2012). Identification of living victims of manual strangulation by MR imaging of the neck.

[9] Gascho D, Heimer J, Thali MJ, Flach PM (2020). The value of MRI for assessing danger to life in nonfatal strangulation. Forensic Imaging.

[10] Bruguier C, Genet P, Zerlauth JB (2020). Neck-MRI experience for investigation of survived strangulation victims. Forensic Sci Res.

[11] Heimer J, Tappero C, Gascho D (2019). Value of 3T craniocervical magnetic resonance imaging following nonfatal strangulation. Eur Radiol.

[12] Ogris K, Widek T, Pivec SM (2013). Comparison of MRI of the neck with external findings in survived manual strangulation.

[13] Bolliger S, Bollmann M, Eisenhart D et al (2012) Schädigung durch Strangulation. Available from: https://www.sgrm.ch/inhalte/Forensische-Medizin/Strangulation_final_rev.pdf

[14] Yen K, Vock P, Christe A (2007). Clinical forensic radiology in strangulation victims: forensic expertise based on magnetic resonance imaging (MRI) findings. Int J Legal Med.

[15] Plattner T, Bolliger S, Zollinger U (2005). Forensic assessment of survived strangulation. Forensic Sci Int.

[16] De Boos J (2019). Review article: non-fatal strangulation: hidden injuries, hidden risks. Emerg Med Australas.

[17] Christe A, Thoeny H, Ross S (2009). Life-threatening versus non-life-threatening manual strangulation: are there appropriate criteria for MR imaging of the neck?. Eur Radiol.

[18] Christe A, Oesterhelweg L, Ross S (2010). Can MRI of the neck compete with clinical findings in assessing danger to life for survivors of manual strangulation? A statistical analysis. Legal Med (Tokyo Japan).

[19] Ruder TD, Gonzenbach A, Heimer J (2023). Imaging of alert patients after non-self-inflicted strangulation: MRI is superior to CT. Eur Radiol.

[20] Bornik A, Heinze S, Campana L (2019). Theoretische Grundlagen Der Forensischen Bildgebung: Arbeitsgemeinschaft Forensische Bildgebung (AGFB) Der Deutschen Gesellschaft für Rechtsmedizin (DGRM). Rechtsmedizin.

[21] Heimer J, Arneberg L, Blunier S (2023). Under-reporting of forensic findings: craniocervical emergency imaging in cases of survived hanging. Forensic Sci Med Pathol.

[22] Rost T, Kalberer N, Scheurer E (2017). A user-friendly technical set-up for infrared photography of forensic findings. Forensic Sci Int.

[23] Arbeitsgruppe Forensische Bildgebung, SGRM (2018) Forensische Infrarotfotographie

[24] Möller TB, Reif E (2019) Taschenatlas Schnittbildanatomie, 2., überarbeitete Auflage. Thieme Verlag

[25] Landis JR, Koch GG (1977). The measurement of observer agreement for categorical data. Biometrics.

[26] Matusz EC, Schaffer JT, Bachmeier BA (2020). Evaluation of Nonfatal Strangulation in Alert adults. Ann Emerg Med.

[27] Marty M, Dobay A, Ebert L et al (2022) MRI segmentation of cervical muscle volumes in survived strangulation: is there an association between side differences in muscle volume and the handedness of the Perpetrator? A retrospective study. Diagnostics (Basel) 12. 10.3390/diagnostics12030743 10.3390/diagnostics12030743 PMC8947368 35328295

[28] Hassler EM, Ogris K, Petrovic A (2015). Contrast of artificial subcutaneous hematomas in MRI over time. Int J Legal Med.

[29] Pan P, Xu L, Zhang H et al (2020) A review of hematoma components clearance mechanism after subarachnoid hemorrhage. Front NeuroSci 14 10.3389/fnins.2020.00685 PMC7358443 32733194

[30] Tran AP, Warren PM, Silver J (2018). The Biology of Regeneration failure and success after spinal cord Injury. Physiol Rev.

[31] Dunn RJ, Sukhija K, Lopez RA (2023). Strangulation injuries.

[32] Funk M, Schuppel J (2003). Strangulation injuries. WMJ.

[33] Sharman LS, Fitzgerald R, Douglas H (2023). Medical evidence assisting non-fatal strangulation prosecution: a scoping review. BMJ Open.

[34] Alessandrino F, Keraliya A, Lebovic J (2020). Intimate Partner violence: a primer for radiologists to make the invisible. Visible RadioGraphics.

[35] Pollanen MS, Chiasson DA (1996). Fracture of the hyoid bone in strangulation: comparison of fractured and unfractured hyoids from victims of strangulation. J Forensic Sci.

[36] Smith SM (2002). Fast robust automated brain extraction. Hum Brain Mapp.

